# The effect of developmental nutrition on life span and fecundity depends on the adult reproductive environment in *Drosophila melanogaster*

**DOI:** 10.1002/ece3.1389

**Published:** 2015-02-18

**Authors:** Christina M May, Agnieszka Doroszuk, Bas J Zwaan

**Affiliations:** Laboratory of Genetics, Plant Sciences, Wageningen UniversityWageningen, 6708 PB, the Netherlands

**Keywords:** Aging, developmental constraints, *Drosophila melanogaster*, life history, predictive adaptive response, reproduction, thrifty phenotype

## Abstract

Both developmental nutrition and adult nutrition affect life-history traits; however, little is known about whether the effect of developmental nutrition depends on the adult environment experienced. We used the fruit fly to determine whether life-history traits, particularly life span and fecundity, are affected by developmental nutrition, and whether this depends on the extent to which the adult environment allows females to realize their full reproductive potential. We raised flies on three different developmental food levels containing increasing amounts of yeast and sugar: poor, control, and rich. We found that development on poor or rich larval food resulted in several life-history phenotypes indicative of suboptimal conditions, including increased developmental time, and, for poor food, decreased adult weight. However, development on poor larval food actually increased adult virgin life span. In addition, we manipulated the reproductive potential of the adult environment by adding yeast or yeast and a male. This manipulation interacted with larval food to determine adult fecundity. Specifically, under two adult conditions, flies raised on poor larval food had higher reproduction at certain ages – when singly mated this occurred early in life and when continuously mated with yeast this occurred during midlife. We show that poor larval food is not necessarily detrimental to key adult life-history traits, but does exert an adult environment-dependent effect, especially by affecting virgin life span and altering adult patterns of reproductive investment. Our findings are relevant because (1) they may explain differences between published studies on nutritional effects on life-history traits; (2) they indicate that optimal nutritional conditions are likely to be different for larvae and adults, potentially reflecting evolutionary history; and (3) they urge for the incorporation of developmental nutritional conditions into the central life-history concept of resource acquisition and allocation.

## Introduction

Nutrition is a primary determinant of life span, the rate of aging, and reproductive capacity (Weindruch and Walford 1982; Chippindale et al. 1993; Good and Tatar 2001; Walker et al. 2005; Fontana et al. 2010), and as such, its relationship to life history has been studied extensively. The bulk of this research has focussed on the impact of adult nutritional quantity and quality, leading to important insights into the field of gerontology. For instance, the discovery of life span extension upon dietary restriction across many different animal species has resulted in a booming field concerned with characterizing the mechanism and specific nutrient dependencies of the effect (Weindruch and Walford 1982; Austad 1989; Chippindale et al. 1993; Grandison et al. 2009). However, a growing body of evidence suggests that developmental nutrition can also impose far-reaching effects on adult traits, including life span and fecundity (Gluckman and Hanson 2004; Boggs and Freeman 2005; Brakefield et al., 2005; Cleal et al. 2007; Gluckman et al. 2008; Barrett et al. 2009; Joy et al. 2010; Dmitriew and Rowe 2011).

Twenty-five years ago, Barker et al. (1989) found that human infants with low birth weights had higher adult mortality from cardiovascular disease. In this case, low birth weight was regarded as a proxy for malnutrition in utero. This finding has since been confirmed in many other epidemiological studies, which have tied undernutrition in utero to an increased risk of traits associated with the metabolic syndrome – a disorder of energy storage which increases the risk of heart disease and type II diabetes (Barker et al. 1989; Leon et al. 1998). In mammalian models for these observations, either inadequate or excessive developmental nutrition has been shown to increase the incidence of traits of the metabolic syndrome, including decreased glucose tolerance, obesity, and diabetes (Painter et al. 2005; George et al. 2012; Barker and Thornburg 2013). In some cases, this has also resulted in increased mortality rates (Aihie Sayer et al. 2001; Ozanne and Hales 2004). In order to interpret these effects in relation to ecological and evolutionary theory (Van den Heuvel et al. 2013), and to quantify the epidemiological consequences for health, the effects of variation of the developmental environment in concert with the adult environment should be assessed. However, given the long life span and cost of upkeep of mammalian models, large factorial designs considering multiple life-history traits across different environments quickly become infeasible.

Studies using more tractable insect models have shown that poor nutrition during development generally results in detrimental fitness effects including decreased size, fecundity, and life span (Zwaan et al. 1991; Kaspi et al. 2002; Boggs and Freeman 2005; Blanckenhorn 2006; Barrett et al. 2009; Bauerfeind et al. 2009; Colasurdo et al. 2009; Kolss et al. 2009; Zajitschek et al. 2009; Dmitriew and Rowe 2011). In fact, it is often assumed that poor larval food inevitably leads to detrimental effects in the adult. Several recent studies, however, suggest that the effect of the developmental environment depends on the specific adult environment experienced (Allen and Marshall 2013). For example, Adler et al. (2013) highlighted the context dependence of the effect of larval food on adult life span in the neriid fly *Telostylinus angusticolus* – when housed in same-sex groups, males raised on calorically rich larval food lived longer than females; however, this difference disappeared in mixed-sex groups. A similar interaction with housing conditions was shown for adult nutrition, where the extent of life span changes in response to nutrition in male fruit flies depended on whether or not the flies were kept in mixed-sex groups (Zajitschek et al. 2013). Because increasing reproduction often comes at the expense of life span (Harshman and Zera 2007; Kenyon 2010; Dmitriew and Rowe 2011), it is important to know how nutritional manipulations affect longevity in environments with differing reproductive potentials (i.e., the extent to which females can reach their full reproductive potential). Indeed, the reproductive potential of the environment, and the differing costs associated with achieving that potential after development on foods differing in quality as larvae, might be the driving force behind some of the interactions between larval and adult nutritional environment.

Mechanistic links between diet and aging have often been explored using *Drosophila melanogaster* as a model organism (Mair et al. 2005; Min et al. 2007). To our knowledge, only one study has addressed the effect of developmental nutrition on both adult longevity *and* fecundity in *Drosophila*. It is important to know how both of these traits respond as aging is characterized both by accelerating mortality rates with time and by an associated decline in offspring production (Kirkwood and Rose 1991; López-Otín et al. 2013). Tu and Tatar (2003) deprived third instar larvae of yeast and found that they displayed decreased fecundity but no concomitant change in longevity as adults. Applying yeast deprivation to third instar larvae only, however, is likely to cause different effects compared to limitation across the whole developmental period (Danielsen et al. 2013), a methodology more comparable to approaches taken in other species when evaluating the effects of adult nutrition. It is also important to note that research in insects on developmental nutrition concerns primarily the effects of underfeeding, and the effects of overfeeding are less well-known, although it has been shown in mammals that the effects of over- and underfeeding could be phenotypically similar (Ford and Long 2011).

**Figure 1:**
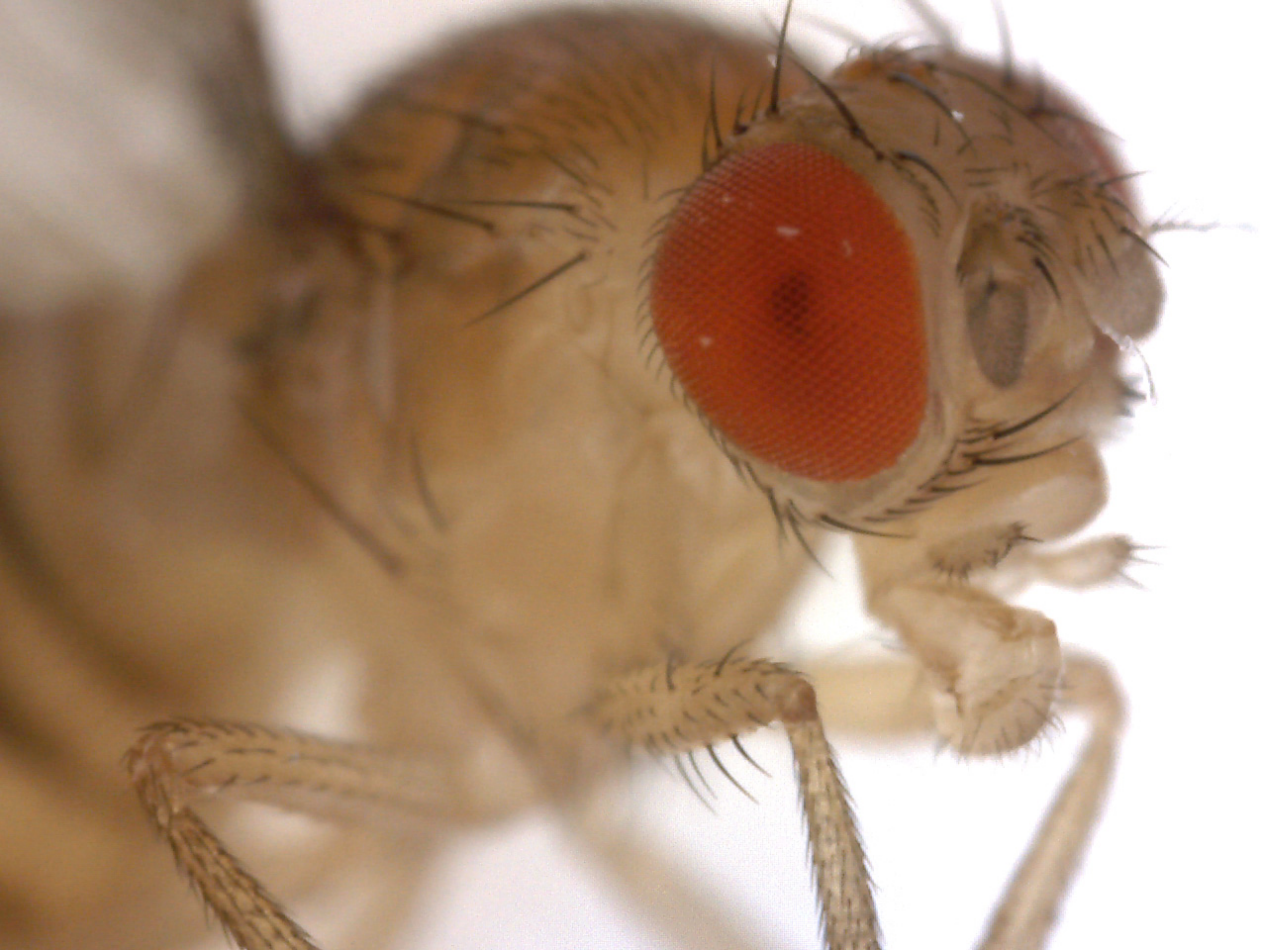
*Drosophila melanogaster*.

In this study, we address the effect of under- and overnutrition of *D. melanogaster* (Fig.1) during the entire juvenile stage on longevity, fecundity, and other life-history traits. We combine these larval nutritional manipulations with three adult reproductive environments (singly mated, SM; singly mated with yeast, SMY; and continuously mated with yeast, CMY) in a full-factorial design in order to determine whether adult environment modulates the effects of developmental nutrition. Generally, the addition of yeast increases fecundity in *Drosophila* (Bass et al. 2007), while the presence of a male allows females to reach higher reproductive potentials by preventing sperm depletion (Kaufman and Demerec 1942), despite shortening life span (Partridge et al. 1987). We hypothesize that the detrimental effects of developmental under- or overnutrition will be highest in the most reproductively conducive adult environment, as presumably both the under- and overfed flies are not able to make full use of the reproductive potential of the environment or will pay a greater cost in terms of life span for doing so.

## Materials and Methods

### Drosophila stock and culturing

The stock population originated from wild populations collected in 2006 from six locations across Europe. To ensure that the genetic variation of the original wild population was equally represented in the stock, we performed four rounds of crosses among the six component populations (Data S1), ensuring that the effects of developmental nutrition are unlikely to be genotype specific. The stock population has been maintained in the laboratory for more than forty generations under standard laboratory conditions (25°C, 65% humidity, 12 h:12 h light:dark cycle, 14-day generation time, and a standard control diet (1×) of 70 g yeast (Fermipan Instant Yeast Red Label), 100 g sugar, 20 g agar, 15 mL nipagin, and 3 mL propionic acid per liter of water) at a population size of approximately 2,000 individuals.

### Larval diet

Eggs collected from 4-day-old adults of the stock population were transferred to vials filled with 7 mL of media (100 eggs/vial, 75-mm vial diameter). Larvae were raised on media where yeast and sugar content was manipulated to obtain diet treatments representing poor, control, and rich food levels. The concentrations of yeast and sugar were relative to those of the standard medium: we used 0.25× concentration for the poor food, 1× for control, and 2.5× for the rich food treatments (Table S1). Amounts of agar, nipagin, and propionic acid remained unchanged across all food levels.

### Experimental setup

Two cohorts of flies were raised on the three larval food levels. In the first cohort, development time, survival from egg to adult, larval weight, adult weight, egg weight, and virgin survival were assessed. In this cohort, all adult flies were maintained on the control medium. In the second cohort, female survival and fecundity were assessed in three adult reproductive environments: singly mated flies on control medium (SM), singly mated flies on control medium with yeast supplement (SMY), and continuously mated flies on control medium with yeast supplement (CMY). The full-factorial setup in the second cohort allowed for the estimation of the relative importance of developmental food conditions and adult reproductive environment on life span and reproduction.

### Developmental time and larval survival

Developmental time and larval survival were assessed for 400 individuals per food level (four vials of 100 eggs each). The number of newly eclosed flies was recorded every hour between 8:00 am and 5:00 pm from the first day of eclosion until no new flies had eclosed for more than 5 h.

### Larval weight, adult weight, and egg weight

Larvae were extracted from the medium 4 days after egg-laying following Bochdanovits and de Jong (2003) and weighed in groups of three (*n* = 15) to obtain both wet (fresh) and dry weights (dried for 24 h at 65°C).

Adult flies were weighed in unisex groupings of three individuals, 1 day after eclosion. Weight was measured for 48 flies per treatment (12 groups of three flies each). After wet weight was obtained, flies were dried in an oven at 65°C for 72 h and then re-weighed to obtain dry weight.

After development on the different larval foods, adult females were maintained as virgins on control food for 2 days at a density of 10 females per vial. They were then placed on agar plates with yeast to stimulate egg-laying for 3 h. Eggs were collected and weighed on a Sartorius ultra-microbalance in groups of 20 per larval food (*n* = 8) to obtain wet weight, then dried for 24 h at 65°C in an oven, and re-weighed to obtain dry weight.

All weights were obtained with a Sartorius ultra-microbalance accurate to the nearest 0.1 *μ*g.

### Virgin survival

To measure adult virgin survival, flies were sexed under mild CO_2_ anesthesia between the third and fourth hour after eclosion. Emergence of flies was synchronized by staggering egg collection days. Flies were maintained in unisex groups of five individuals per vial, per sex, and per larval food level (*n* = 20) and transferred to fresh media weekly. Survival was recorded every second day.

### Reproduction and survival of mated flies

After eclosing, flies raised on different larval treatments were kept separately in mixed-sex groups in 250-mL bottles with standard medium for 48 h to allow time for mating. Flies were then sexed under mild CO_2_ anesthesia, and females were transferred to one of three adult treatments: singly mated (SM), singly mated with yeast (SMY), or continuously mated with yeast (CMY). The yeast supplement consisted of 20–30 grains of yeast added to the surface of the medium. Females were housed individually or with a single male and transferred to fresh medium every second day. At this time survival was scored, yeast supplement was reapplied and any dead males were replaced by individuals from the same cohort. Previously inhabited vials were retained until the eggs developed into adults. These offspring were counted, giving an accurate measure of realized female fecundity. This regimen was maintained until all females had died.

### Statistical analysis

#### Cohort 1

Wet and dry weights of eggs, larvae, and adults were analyzed using ANOVAs with larval food as the independent variable. Post hoc determination of differences between larval food treatments was performed using the Tukey HSD test. Survival from egg to adult was analyzed as binomial data with a generalized linear mixed-model approach designating larval food as a fixed factor and vial as a random variable nested within the food treatment.

Egg to adult development time and adult virgin survival were analyzed using Cox proportional hazards with larval food treatment as the independent variable.

#### Cohort 2

Adult mated survival was analyzed using Cox proportional hazards, with larval food treatment and adult reproductive environment as independent variables.

Fecundity was analyzed using a repeated-measures ANOVA which estimated both between- and within-subjects effects. Between-subjects effects address the effect of larval food and adult reproductive environment on total fecundity, while within-subjects effects assess whether larval food or adult reproductive environment affect patterns of reproduction over time. To disentangle significant interactive effects in the model between time and adult reproductive environment, we performed independent ANOVAs for each 48-h period for each adult reproductive environment. The Bonferroni correction was used for post hoc testing (Holm 1979).

Statistical analysis was performed in JMP statistical software (v.9.0.0) and in R (v. 3.0.1) (R Core Team 2013).

## Results

### Effects of larval nutrition on larvae and young adults

**Figure 2:**
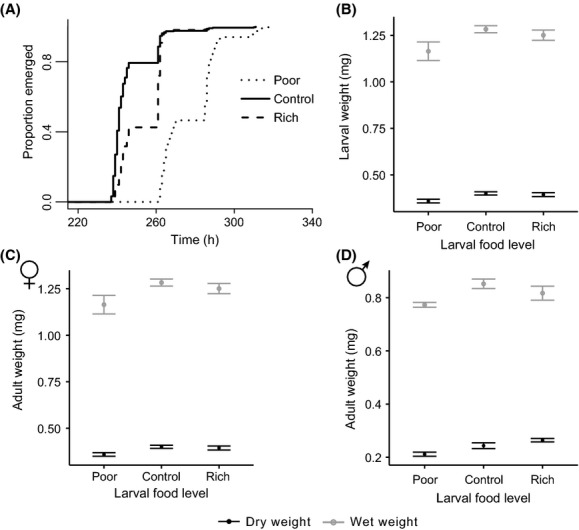
The effect of larval food level on development time (A), larval wet and dry weights (B), adult female wet and dry weights (C), and adult male wet and dry weights (D). All error bars are standard errors of the mean (SE).

Larvae raised on poor and rich food showed a delay in development compared to the control food level (Cox PH model: *χ*^2^ = 553.164, *P* < 0.0001, Fig.2A). While for the larvae raised on rich media, the delay was only about 8 h, larvae on poor food took 34 h longer to develop on average (Fig.2A). In addition, larvae feeding on rich and poor food showed significantly lower survival compared to the control treatment (generalized linear model, best-fit model: AIC = 1014, *z* = 3.42, *P* = 0.00063, mean survival from egg to adult ± SEM: poor = 80 ± 3%, control = 89 ± 1%, rich = 80 ± 3%).

Larval weight at 4 days after hatching was strongly affected by larval food level (ANOVA: *F*_2,42_ = 56.6690, *P* < 0.0001 and *F*_2,42_ = 59.4345, *P* < 0.0001 for wet and dry weights, respectively, Fig.2B). Both poor food and rich food raised larvae were lighter than control larvae (Tukey HSD: *P* < 0.001 for poor and rich raised larvae, respectively, Fig.2B); however, the effect was much stronger on poor food raised larvae, which were 65% lighter than controls.

Both male and female flies developing on a poor food diet weighed significantly less as adults than those raised on control and rich larval food (ANOVA: *F*_2,78_ = 9.641, *P* = 0.006 and *F*_2,70_ = 21.273, *P* < 0.001 for wet and dry weights, respectively; Fig.2C and D), which means that the longer period of larval growth did not compensate entirely for the adverse effects of poor food on body mass. Interestingly, there was no difference in adult size between flies raised on rich and control food levels (Tukey HSD: *P* = 0.907 and *P* = 0.277 for wet and dry weights, respectively, Fig.2C and D), indicating that flies raised on rich food were able to compensate for their larval weight differential, perhaps via their slightly increased development time. It is worth noting though, that this compensation may not have been complete, as there still appears to be a trend toward lower weight in rich-raised flies. Both sexes showed similar responses to larval food (ANOVA: *F*_2,78_ = 0.332, *P* = 0.72 and *F*_2,70_ = 0.949, *P* = 0.392 for wet and dry weights, respectively), and, as expected, females were heavier than males, irrespective of larval food conditions (ANOVA: *F*_1,78_ = 314.883, *P* < 0.0001 and *F*_1,70_ = 347.38, *P* < 0.0001 for wet and dry weights, respectively).

Eggs laid by females raised on poor food had a higher wet weight than those of other treatments (ANOVA: *F*_2,21_ = 4.253, *P* = 0.0281, mean wet weight (mg) ± SEM: poor = 0.220 ± 0.003, control: 0.165 ± 0.014, rich: 0.2006 ± 0.018); however, no difference was observed when the eggs were dry (ANOVA: *F*_2,21_ = 1.322, *P* = 0.288, mean dry weight (mg) ± SEM: poor = 0.055 ± 0.004, control: 0.047 ± 0.003, rich: 0.055 ± 0.003), indicating a similar resource investment in egg production. Overall, while our results show that larvae and young adults suffer what have classically been considered detrimental effects of poor nutrition such as increased development time and decreased adult weight, the effects of rich nutritional levels are less pronounced.

### Effects of larval nutrition on virgin longevity

**Figure 3:**
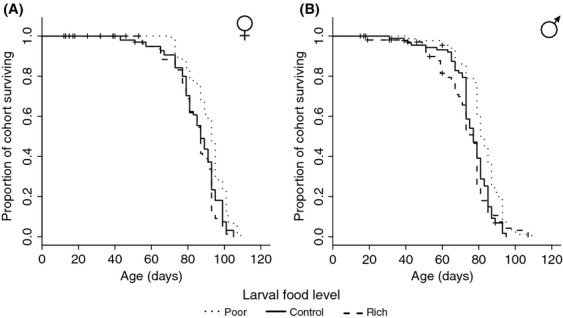
The effect of larval food level on virgin adult female (A) and male (B) survival. Rearing on poor larval food increases longevity of both male and female flies, while flies raised on control and rich food as larvae show similar adult life spans in both sexes.

Females were longer lived than males (Cox PH model: *χ*^2^ = 74.739, *P* < 0.0001), but both sexes showed a similar response to larval treatments. Remarkably, flies raised on poor food actually lived 7 and 8% longer on average than those raised on control and rich medium, respectively (Cox PH model: *χ*^2^ = 28.8517, *P* < 0.0001; Fig.3A and B). This translates into an increase in life span of about 6 days on average, relative to the control, while flies raised as larvae on control and rich food did not differ in life span (*P* = 0.73; Fig.3A and B).

### Effects of larval nutrition and reproductive environment on mated longevity

There was a profound effect of reproductive environment on longevity (Cox PH model: *χ*^2^ = 34.955, *P* < 0.0001, Fig. 5A–C). Longevity decreased stepwise relative to singly mated females; adding yeast decreased longevity by approximately 8% while adding yeast and allowing continuous mating decreased average longevity by approximately 30% (Fig. 5A–C). In contrast to virgin longevity, mated longevity was not influenced by larval food level (Cox PH model: *χ*^2^ = 2.3087, *P* = 0.315).

### Effects of larval nutrition and reproductive environment on reproduction

**Figure 4:**
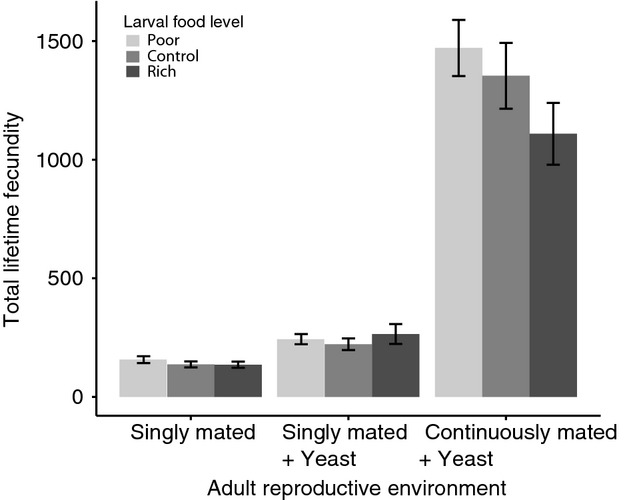
Total lifetime fecundity as a function of larval food level and adult reproductive environment. Allowing continuous mating with added yeast results in significantly higher lifetime fecundity than single mating, or single mating with yeast conditions, regardless of larval food conditions. While there is no significant main effect of larval food on total lifetime fecundity, there is a marginally significant interactive effect between larval and adult conditions due to flies raised on poor larval food having slightly higher lifetime fecundity than rich larval food raised flies when experiencing continuous mating with added yeast conditions (Bonferroni post hoc test, *P* = 0.10).

**Figure 5:**
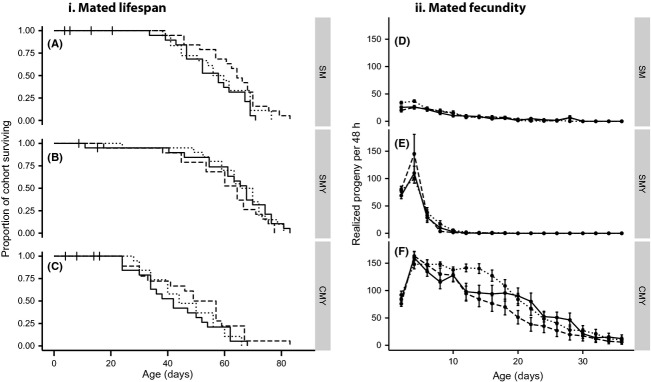
The effect of larval food conditions and adult reproductive environment on mated longevity (panel i) and fecundity over time (panel ii). In contrast to virgin longevity, there are no differences in mated longevity between larval food levels within an adult condition (A–C). However, adult conditions do profoundly affect life span, with life span decreasing dramatically from singly mated (SM) (A) to singly mated with added yeast (SMY) (B) to continuously mated with added yeast conditions (CMY) (C). Maximum reproductive rate and total reproduction occur when flies were continuously mated with yeast (F), while adding yeast alone (E) only increases maximum reproductive rate but not total reproduction relative to single mated flies (D). In addition, flies raised on poor larval food have higher early reproduction when singly mated (D) and higher mid-reproductive span reproduction when continuously mated with yeast (F) relative to flies raised on rich food

The adult reproductive environment profoundly affected both total reproduction (Table1, Fig.4) and patterns of reproduction over time (Table2, Fig.5D–F). The strongest effect was seen when flies were continuously mated with added yeast (CMY) – this treatment resulted in much higher lifetime reproduction (nine and six times higher than females experiencing single mating or single mating plus yeast conditions, respectively; Bonferroni post hoc test: *P* < 0.001 for both, Fig.5D–F). CMY also increased the maximum rate of reproduction achieved per 48-h period, with average fecundity from ages 2 to 4 days of 161.54 ± 4.76 SE relative to SMY (109.41 ± 6.37) and SM (29.38 ± 1.83) (ANOVA days 2 to 4: *F*_2,162_ = 188.43, *P* < 0.001). In addition, CMY flies continued reproducing until day 36 of adult life, while in SMY and SM flies, all reproduction had ceased by days 20 and 26, respectively (Fig.5D–F). In contrast, the difference between SM and SMY was subtler; while they did not differ in total lifetime fecundity (Bonferroni post hoc test: *P* = 0.43, Fig.4), their patterns of reproduction across life were different. (Fig5D–F) Adding yeast to singly mated females resulted in a rapid burst of reproduction early in life followed by a quick (near) cessation of reproduction (Fig.5E). In contrast, singly mated females without yeast did not reach a similarly high peak of early reproduction, but their reproduction was spread out across the life span (Fig.5D).

**Table 1:** Tests of between-subjects effects on overall fecundity across the life span

Source of variation	df	Sum of squares	Mean square	*F*	*P* value (Greenhouse–Geisser)
Larval food level (LFL)	2	25,608	12,804	2.502	0.085
Adult reproductive environment (ARE)	2	2,963,847	1,481,923	289.531	<0.001
LFL × ARE	4	46,088	11,522	2.251	0.066
Error	161	824,055	5118		

**Table 2:** Tests of within-subjects effects on patterns of reproduction over time

Source of variation	df	Sum of squares	Mean square	*F*	*P* value (Greenhouse–Geisser)
Time	5.357	2,010,850	375,383	223.964	<0.001
Time × larval food level (LFL)	10.714	37,545	3504	2.091	0.02
Time × adult reproductive environment (ARE)	10.714	1,319,910	123,200	73.504	<0.001
Time × LFL × ARE	21.427	67,144	3134	1.870	0.01
Error (time)	862.44	1,445,533	1676		

There was also a near significant interactive effect between the larval food environment and adult reproductive environment on total fecundity (Table1, Fig.4), as flies raised on poor food had slightly higher lifetime reproduction than those raised on rich food in the CMY condition (Bonferroni post hoc test, *P* = 0.10). Indeed, contrary to expectation, flies raised on the lowest food as larvae did not show compromised reproduction in any adult reproductive environment.

In addition, larval food modified patterns of reproduction across time depending on the adult reproductive environment (Table2, Fig.5D–F). In order to break down this interaction, we performed individual ANOVAs per adult reproductive environment on each 48-h time period in which reproduction was measured. This showed that under singly mated conditions, females raised on poor food had higher early reproduction than those raised on rich food (days one to four of adult life, Table3), while control and rich-raised flies did not differ. For the rest of the life span, the rate of reproduction of poor raised flies was similar to the other larval treatments. When flies were singly mated with yeast, the differences between larval food levels in patterns of reproduction across the life span disappeared (repeated-measures ANOVA: *F*_2.5,137.4_ = 0.749, *P* = 0.588). However, when under the CMY (continuous mating + yeast) condition, again poor flies showed an increase in reproduction relative to flies raised on rich food, while control and rich-raised flies did not differ from each other. Notably, the increase in fecundity of poor-raised flies appeared later on in life, from days 12 to 16 of life (Table3). Thus, it appears that not only does larval food alter patterns of reproduction, and therefore the adult life history of the fly, but this effect is also dependent on the adult reproductive environment.

**Table 3:** ANOVAs of total fecundity per time point and adult reproductive environment. Only significant results shown

Age (days)	Adult reproductive environment	ANOVA results			Means ± SE			Post hoc tests		
		*F*	df	*P*	Poor	Control	Rich	Poor vs. Control	Poor vs. Rich	Control vs. Rich
2	Singly mated	6.48	2,53	0.003	34.1 ± 2.8	25.1 ± 2.6	19.7 ± 3.1	0.074	0.002	0.373
	Continuous + yeast	4.33	2,53	0.018	96.7 ± 5.5	84.5 ± 3.1	80.3 ± 3.2	0.0957	0.0182	0.7467
4	Singly mated	4.80	2,53	0.012	37.1 ± 2.5	25.8 ± 2.9	25.6 ± 3.4	0.0262	0.0239	0.9992
12	Continuous + yeast	4.03	2,53	0.024	141.4 ± 7.1	102.9 ± 15.4	93.1 ± 14.3	0.0885	0.0267	0.8501
14	Continuous + yeast	5.61	2,53	0.006	140.2 ± 8.9	95.8 ± 13.9	83.3 ± 14.6	0.0400	0.0071	0.7667
16	Continuous + yeast	3.64	2,53	0.033	127.2 ± 10.3	93.9 ± 15.5	76.3 ± 14.3	0.1956	0.0282	0.6303

## Discussion

### Effects of larval nutrition on larvae and young adults

A wide array of insect literature has found that calorically poor food during development leads to increased development time and decreased adult weight (Kaspi et al. 2002; Colasurdo et al. 2009; Kolss et al. 2009; Dmitriew and Rowe 2011), which agrees with our finding that developing on poor food decreases larval and adult weight while increasing development time. Although the literature on overfeeding during development in insects is rather sparse, existing studies suggest that high-protein diets accelerate larval development, while high sugar levels can cause growth inhibition and development of “hallmarks” of type 2 diabetes (Pasco and Léopold 2012; Danielsen et al. 2013). In our study, a high-protein–high-sugar diet resulted in a moderate increase of development time and a decrease of larval but not adult weight. It appears that flies raised on rich developmental nutrition may use an increase in development time to overcome the challenges of overfeeding, ultimately resulting in flies phenotypically indistinguishable from control flies in all other adult traits we assayed, but with lower fecundity than poor-raised flies at certain ages.

As found previously by Prasad et al. (2003) and Vijendravarma et al. (2010), flies raised on poor food as larvae laid significantly heavier eggs than those raised on control food, despite being smaller adults. Vijendravarma et al. (2010) hypothesized that this was due to enhanced maternal egg provisioning. Our results showed that the increased egg weight in poor-raised females was due to increased water content of the eggs; the dry weight of eggs did not differ between the control and poor raised females. This increased water content could be caused simply by increased allocation of water by the poor raised flies, or by a change in the allometry of the different components of the egg. Whether either of these mechanisms is beneficial to the offspring is unclear, but merits further testing.

### Effects of larval nutrition on virgin longevity

While several traits responded as expected to larval nutrition, virgin longevity was a notable exception in our experiment. Flies raised on rich food as larvae showed no difference relative to control in terms of life span, while flies of both sexes raised on poor food displayed a 7% increase in life span relative to control (Fig2A and B). While this increase may seem rather modest, it is by no means negligible and indicates a far-reaching effect of larval nutrition on life span. In fact, this extension falls within the range of life span extension achieved by induction of dFOXO (Hwangbo et al. 2004), a key gene in the insulin-signaling pathway (Giannakou and Partridge 2007).

Of the few studies that have applied restricted nutrition to *Drosophila* during development and consequently measured longevity, only one has shown an increase in life span. Zwaan et al. (1991) found that adult life span was increased in flies that had been transferred as larvae onto agar-only medium after 60 h of development. In contrast, Tu and Tatar (2003) found that removing yeast in the third instar did not affect adult longevity. It seems rather likely that reduction of both yeast and sugar, as done by Zwaan et al. (1991) and in our experiments, would have considerably different effects to reducing only yeast. In fact, for adult *Drosophila,* it has been shown that the ratio of carbohydrate to protein (i.e., sugar to yeast) is often very important in determining adult longevity (Lee et al. 2008). In addition, studies on *Drosophila* larvae have indicated that different relative protein and sugar content of developmental food can cause long-term alterations in insulin signaling with possible effects on adult traits (Pasco and Léopold 2012; Danielsen et al. 2013). In these studies, high-sugar diets induced delayed eclosion, smaller body size, and a type 2 diabetes-like phenotype in adults. In the Tu and Tatar (2003) study, the lack of any protein in the diet but ample sugar caused several of those effects, but the demographic patterns of aging remained normal. Perhaps decreasing both sugar and yeast in a balanced way, as in our study and that of Zwaan et al. (1991), could induce other types of long-term metabolic changes resulting in a long-lived phenotype. Indeed, body composition (most notably, relative fat content) of the adults was significantly affected by the larval developmental environment in the Zwaan et al. (1991) study.

### Effects of larval nutrition and reproductive environment on mated longevity and reproduction

In contrast to virgin flies, the life span of mated flies was not affected by larval food, regardless of the adult reproductive environment. Interestingly, this closely parallels the response to selection for life span observed by Zwaan et al. (1995), wherein increases in life span were observed in virgin but not mated selected lines. One possible explanation is that the life span shortening effects of reproduction make life span differences more difficult to detect. In mated flies, the adult reproductive environment acted as the main determinant of mated life span and fecundity. This is not a novel finding, in fact, an increase in fecundity with added yeast and added males coupled with a concomitant decrease in longevity is well documented (Kaufman and Demerec 1942; Partridge et al. 1987; Bass et al. 2007). However, these adult reproductive environments were included in our experiment in order to determine whether or not the effect of developmental environment depended on the adult reproductive environment, and in this sense, they proved very instructive. Specifically, we had hypothesized that the negative effects of poor or rich developmental food would be more pronounced in the adult environments in which reproduction was most favoured (added yeast and males). This proved to be incorrect as neither poor or rich raised flies suffered significantly decreased longevities or fecundities relative to the control in either of these situations (Fig.4).

Across insects, adult size is quite strongly correlated with fecundity (Honěk 1993). In a meta-analysis of 68 insect species, Honěk (1993) found that for every one percent increase in body mass, median fecundity increased by 0.95%. In our experiment, flies raised on poor food were 9.3 and 10.8% smaller than rich and control raised flies, respectively (Fig.2C and D). However, at no point did they display decreased fecundity. Rather, when singly mated without yeast, poor raised females had higher reproduction early in life, and when continuously mated with yeast, poor raised females had increased reproduction in the middle of the reproductive span (Table3). No such differences existed in the SMY condition, likely because the high rates of reproduction afforded by the added yeast resulted in consistent sperm depletion across treatments.

The mechanisms responsible for the increased virgin life span and age-specific fecundity of poor raised flies remain speculative. One potential mechanism is by “viability” selection, as flies raised on poor and rich food have significantly lower larval survival than controls. However, this seems unlikely, as despite both treatments resulting in similar larval survival, only the poor raised flies have increased life span. Two more likely alternative mechanisms are stress-response hormesis or the induction of a thrifty phenotype.

Stress-response hormesis refers to the phenomenon whereby exposure to a mild stressor increases future resistance to stress (Gems and Partridge 2008; R Core Team 2013), usually via induction of chaperone proteins such as those involved in heat shock. In fact, in *C. elegans* brief thermal stress increases life span, and the increase is greater the earlier the stress is applied (Olsen et al. 2006). In our experiment, it is possible that decreased nutrition during early development acts as a hormetic, increasing the robustness of the organism. However, this does not appear to hold true for flies raised on rich food; while these larvae do show some indicators of stress during development, they do not exhibit increased life span or reproduction.

The thrifty phenotype hypothesis (Hales and Barker 1992) proposed that nutritionally poor developmental conditions induce a metabolically thrifty metabolism to survive development, but that this metabolic phenotype can be detrimental later in life. In our case, it is possible that the poor developmental nutrition does indeed impose a change in metabolism, potentially to a more energetically efficient “thrifty” metabolism, while the rich larval food results in the opposite. There is, however, one main difference to the thrifty phenotype hypothesis as proposed by Hales and Barker for humans – humans experiencing poor developmental nutrition show negative consequences of adequate nutrition postutero including an increased risk of developing the metabolic syndrome (Painter et al. 2005; Gluckman et al. 2007; Danielsen et al. 2013). In our flies, this does not appear to be the case. This could be due to differences in the way metabolism influences disease risk in flies as compared to humans, or possibly that the adverse effects in adult flies are induced only in the case of a larval diet dominated by sugars.

In keeping with these hypotheses, one could also consider more proximate explanations of higher reproduction in young adults raised on poor food. A recent study by Aguila et al. (2013) reports that programmed cell death of larval fat cells in the adult is important for female reproduction. The authors report that in 2-day-old adults, more than half of the nutrients acquired by the ovaries are dependent on the death of fat cells and that if programmed cell death is inhibited, ovarian development is delayed. One could imagine that normal levels of programmed cell death in larvae reared on poor food, and therefore, with less larval fat, would result in a higher relative efficiency of programmed cell death in this tissue and consequently facilitate ovarian nutrient acquisition and faster ovarian development. Alternatively, development on poor food could have resulted in adults able to mobilize larval fat to the ovaries more efficiently.

### Relevance for life-history theory: resource acquisition and allocation

Our results indicate that the optimal nutritional conditions for fruit flies differ across the life span. In particular, less nutrient-rich larval diets may be beneficial for adult fitness, at least for females. This may reflect the evolutionary history of this insect in nature, where larval conditions may be substantially poorer on average than the adult ones.

Inspired by observations such as those of Hales and Barker (2001), several adaptive explanations have been put forward. They include a role of the developmental environment as a predictor of the (nutritional) status of the impending adult environment. Natural selection would have favoured genotypes that would adjust their physiological, metabolic, and/or life-history phenotypes to ensure a good match with the early and/or late life adult environment (for instance predictive adaptive responses (Gluckman and Hanson 2004; Gluckman et al. 2007); see also (Van den Heuvel et al. 2013)).

It is tempting to interpret our results in light of such hypotheses. Poor-raised flies have increased early life reproduction when singly mated, which could be an immediate response to an anticipated poor adult environment. As indicated earlier, this effect could have been masked in the added yeast treatment because of additional resource availability and a rapid depletion of sperm in females. Similarly, the increased reproductive output in the CMY-treated flies during midlife may be an indication of increased willingness of poor-raised females to remate and/or an increased allocation during development to reproduction.

The reported effects argue that the role of the larval and adult environment in resource acquisition and allocation should be explicitly incorporated in theoretical models and experimental studies of life-history evolution. Further work to determine the mechanism by which the flies raised on poor food extend their virgin life span and increase fecundity under certain adult conditions can help to understand this response. This could include studies of gene expression, metabolic rate, and stress resistance across the life span to determine whether these are also lastingly affected by developmental environment. In addition, the creation of artificial selection or experimental evolution lines adapted to different larval nutritional environments may help to clarify to what extent the plastic effects of developmental food are adaptive (C. M. May et al. unpubl. data).

## Conclusions

This study shows that while larval overfeeding in *Drosophila* appears to have minimal effects on life span and fecundity, larval underfeeding can dramatically affect life-history traits across a developmental boundary. In addition, this effect depends on the reproductive environment in which the traits are expressed. In contrast to expectation, larval underfeeding extends adult virgin longevity, does not affect mated longevity, and increases fecundity at certain ages. We propose that this could occur in two separate, although not mutually exclusive, ways – either by the induction of stress-response hormesis, producing hardier flies, or via the induction of an altered metabolism which gives the flies a general advantage as adults in these environments. Our results urge for a more explicit incorporation of the developmental environment in life-history theory. Further experiments are suggested to determine the metabolic rate and stress resistance of the flies raised on poor food, as well as to determine potential differences in gene expression between flies raised on poor or rich food. A more clear understanding of the life span advantage gained by development on poor food in the exceedingly tractable model organism *Drosophila* may be instrumental in determining new areas to explore in the human-oriented field of developmental nutrition.

## Data Accessibility

All data used in this manuscript are present in the manuscript and its supporting information.

## Supporting Information

**Data S1.** Derivation of the stock population. **Table S1.** Larval food composition.
