# Supplementary material for: The effect of developmental nutrition on life span and fecundity depends on the adult reproductive environment in Drosophila melanogaster
Source: Ecol Evol. 2015 Feb 18;5(6):1156–68. doi: 10.1002/ece3.1389 (PMC4377260; doi:10.1002/ece3.1389)
Supplement: Supplementary file 1 [file ece30005-1156-sd1.docx]

**Data S1. Derivation of the stock population**

The stock population was derived from 6 wild-caught European populations. The crosses were designed to create a stock population with a high level of genetic polymorphism and to prevent uneven contribution of genetic variation from the initial populations.

Initial component populations:

A: Vienna (Austria; N 48˚ 14.733’; E 016˚ 16.024’)

B: Palic (northern part of Serbia; N 46˚ 05.910’; E 019˚ 45.649’)

C: Predijane (southern part of Serbia; N 42˚ 52.609’; E 022˚ 05.242’)

D: northern Macedonia (N 41˚ 24.290’; E 022˚ 17.951’)

E: Dorjan (southern Macedonia; N 41˚ 20.480’; E022˚ 26.575’)

F: Pournis (Greece; N 39˚ 08.443’; E 023˚ 17.181’)

**Crossing Scheme**

*1. First crossing round: “one to one”*


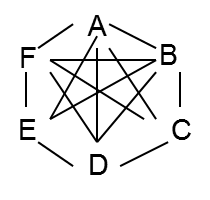


- Populations were crossed with each other in pairs (15 combinations)
- In each cross, 100 females of one component population and 100 males of other component populations were mixed
- Crosses were performed reciprocally (30 crosses in total)
- Crosses deliver “2-genotype populations”: AB, AC, AD, *etc.*


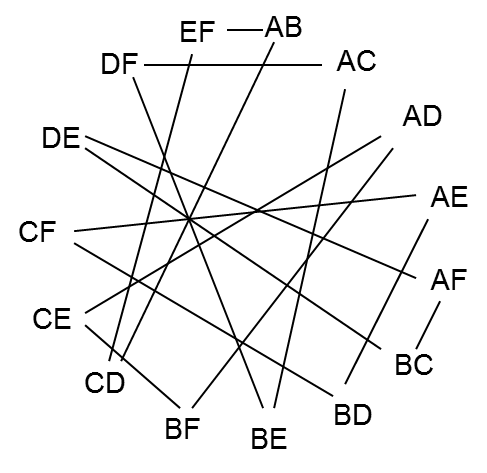
*2. Second crossing round: “each to a different”*

- 2-genotype populations were crossed to other 2-genotype populations derived from different component populations (15 combinations)
- perform reciprocally, 30 crosses
- Crosses deliver “4-genotype populations”: ABCD, ABEF, ADBF, ADCE *etc.*

*3. Third crossing round: “each to a different II”*

- 4-genotype populations were crossed to other 4-genotype populations derived from different component populations
- performed reciprocally, 30 crosses
- Crosses deliver 30 “6-genotype populations”: ABCDEF

*4. Fourth crossing round: “Random mating”*

- 30 “6-genotype populations” ABCDEF were mixed in equal proportions
- The mixed base population was subsequently divided into 4 replicate mixed 6-genotype populations
- The populations were allowed to mate randomly for 3 generations to prevent linkage disequilibrium

**Table S1. Larval food composition**

S1. Diet composition per litre of water

| Diet composition | Low (0.25x) | Control (1x) | High (2.5x) |
| --- | --- | --- | --- |
| Yeast* | 17.5g | 70g | 175g |
| Sugar † | 25g | 100g | 250g |
| Agar | 20g | 20g | 20g |
| Nipagin solution | 15mL | 15mL | 15mL |
| Propionic acid | 3mL | 3mL | 3mL |

*Fermipan Red Label instant yeast

†Suiker Unie Granulated Sugar Extra Fine
